# Aging in Southeast Asia and Japan: Challenges and opportunities

**DOI:** 10.1111/ggi.70062

**Published:** 2025-05-15

**Authors:** Ryota Sakamoto

**Affiliations:** ^1^ Center for Southeast Asian Studies, Kyoto University Kyoto Japan

**Keywords:** 90th percentile age, aging, Japan, older adults, Southeast Asia

## Abstract

Trends in life expectancy and fertility in Southeast Asia reflect the region's vulnerability to conflicts, natural disasters, infectious diseases and other crises. Southeast Asia is characterized by significant diversity, not only in its natural environment, history, religions, economic situations and political systems, but also in its challenges and healthcare systems, which include traditional medicine and local healing practices. Despite these differences, common issues are shared across the region, such as an aging population, the need for universal access to safe and secure healthcare, and the pursuit of peace and environmental harmony. Currently, Japan's aging population is more pronounced than in Southeast Asia, with a notable prevalence of older adults with chronic diseases, including dementia and living alone. However, if current demographic trends continue, most Southeast Asian nations might face similar challenges in the future. This article focuses on the 90th percentile age over time and advocates the importance of considering the relative position of age in society, rather than simply focusing on age alone. Additionally, it is recognized that individuals of the same chronological age can show significant variation in health, shaped by their life circumstances and lifestyles. As Japan leads in addressing aging‐related issues, it plays a critical role in sharing its experiences. Because the health status of certain age groups in society varies from region to region and era to era, depending on the environment and culture, measures should be adapted to the realities of each society, with the cooperation of the international community to address shared challenges. **Geriatr Gerontol Int 2025; 25: 837–854**.

## Graphical abstract

This paper advocates the importance of considering the relative position of age in society, rather than simply focusing on age alone. In Japan, the 90th percentile age has reached 80 years, which means that 10% of the population is aged ≥80 years.

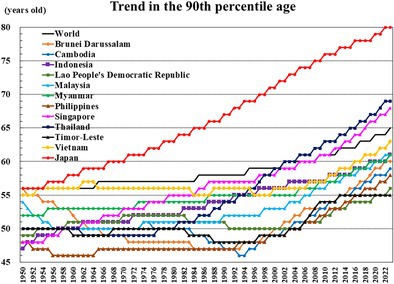

## Introduction

**Figure 1:**
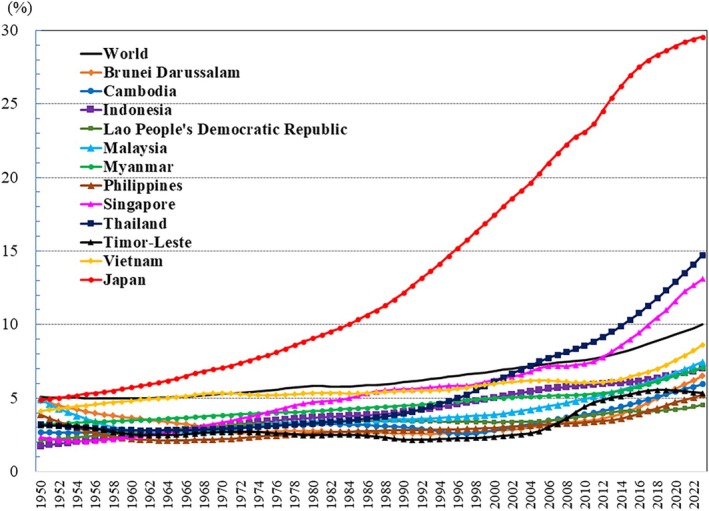
Trend in the percentage of people aged ≥65 years. Data from United Nations, Department of Economic and Social Affairs, Population Division (2024). World Population Prospects 2024, Online Edition.

**Figure 2:**
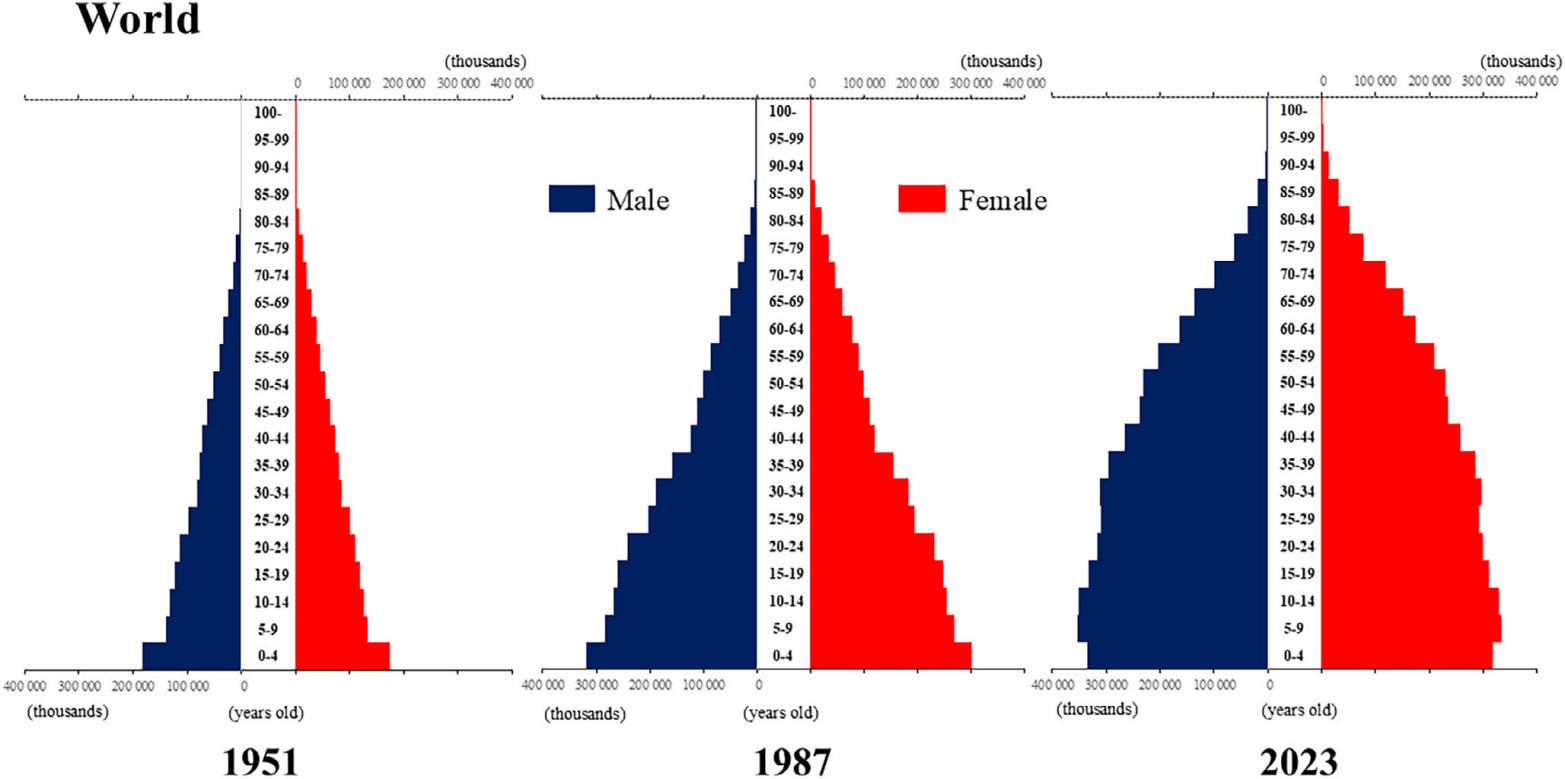
Population pyramid: World. Data from United Nations, Department of Economic and Social Affairs, Population Division (2024). World Population Prospects 2024, Online Edition.

**Figure 3:**
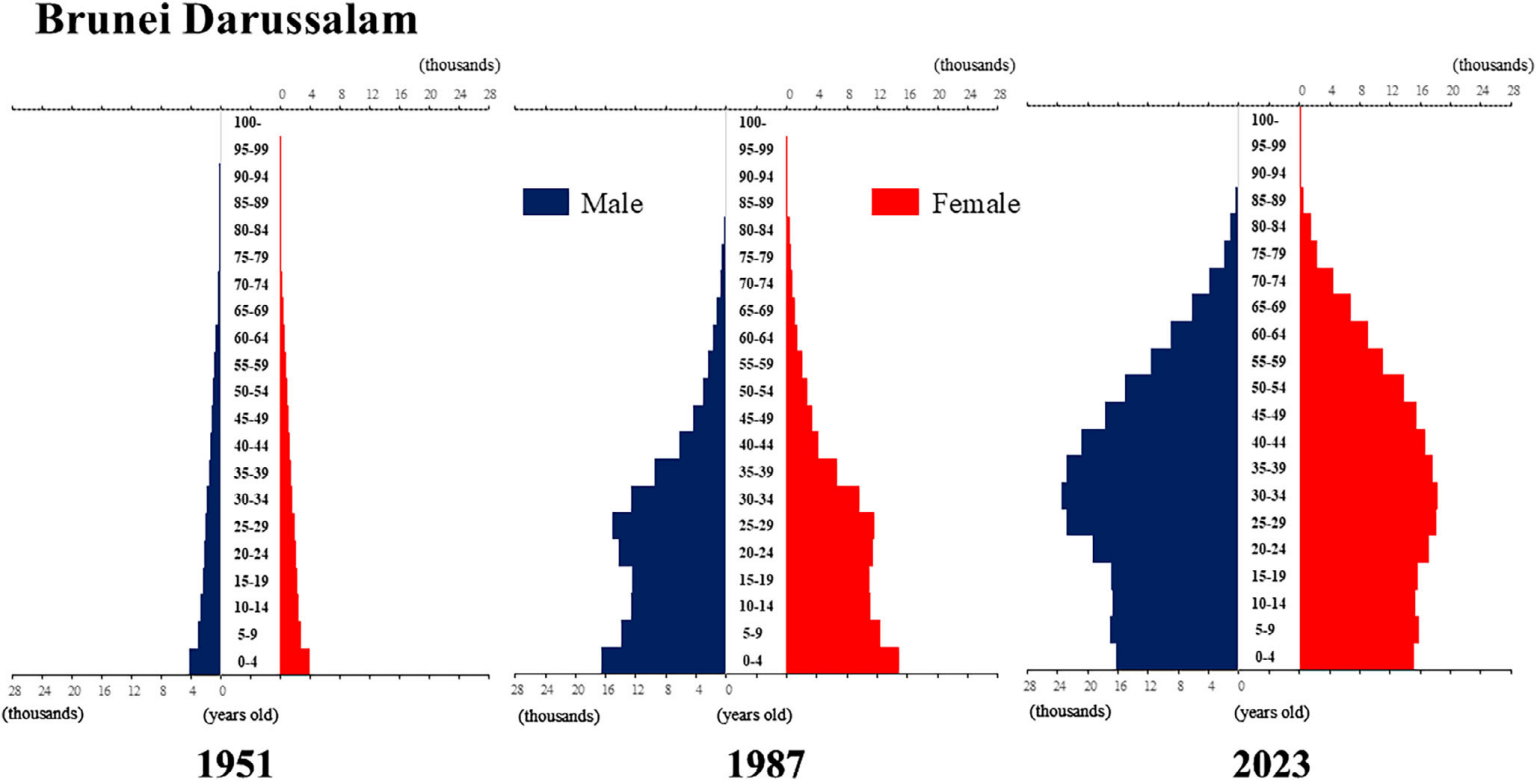
Population pyramid: Brunei Darussalam. Data from United Nations, Department of Economic and Social Affairs, Population Division (2024). World Population Prospects 2024, Online Edition.

**Figure 4:**
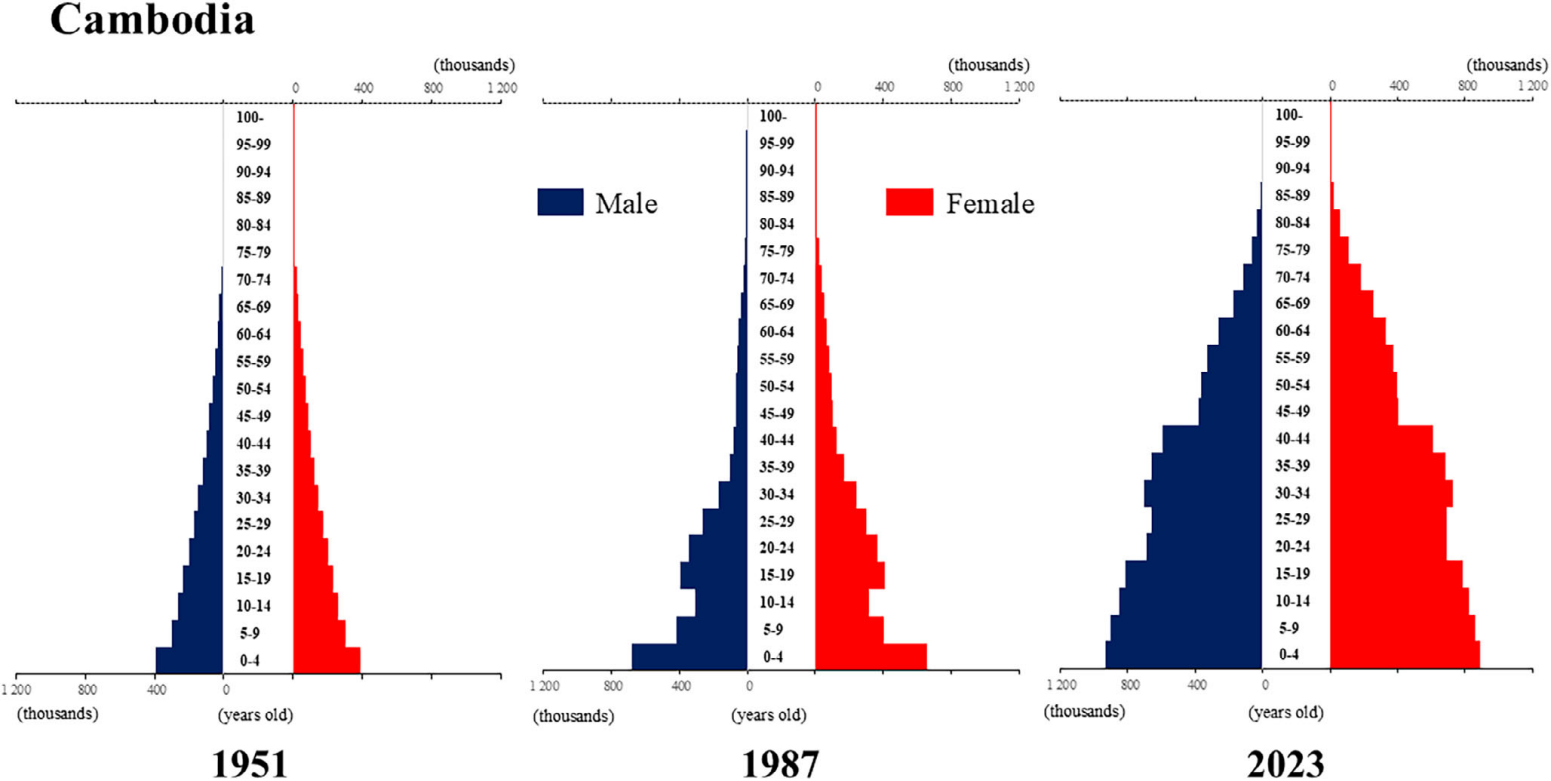
Population pyramid: Cambodia. Data from United Nations, Department of Economic and Social Affairs, Population Division (2024). World Population Prospects 2024, Online Edition.

**Figure 5:**
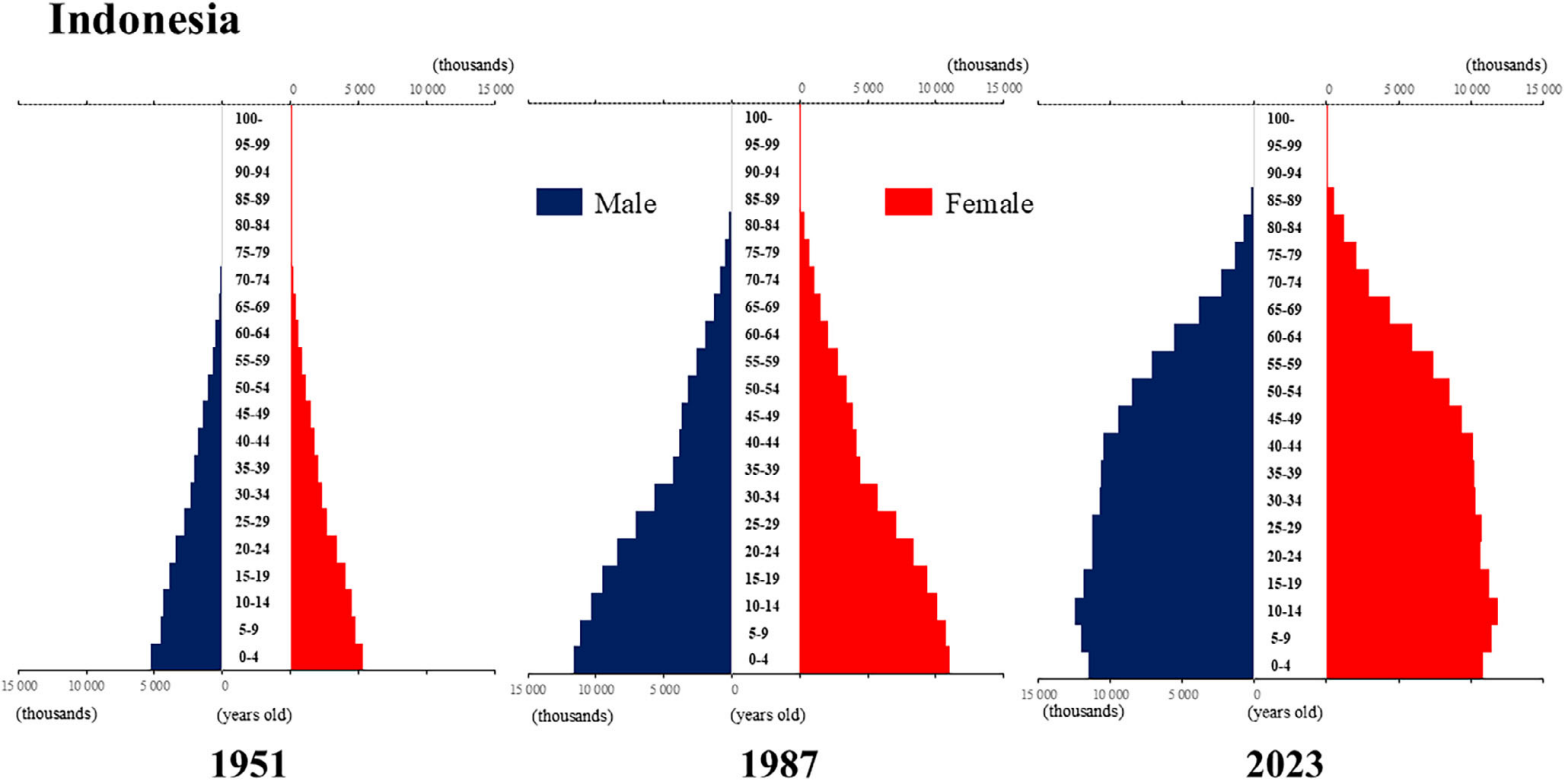
Population pyramid: Indonesia. Data from United Nations, Department of Economic and Social Affairs, Population Division (2024). World Population Prospects 2024, Online Edition.

**Figure 6:**
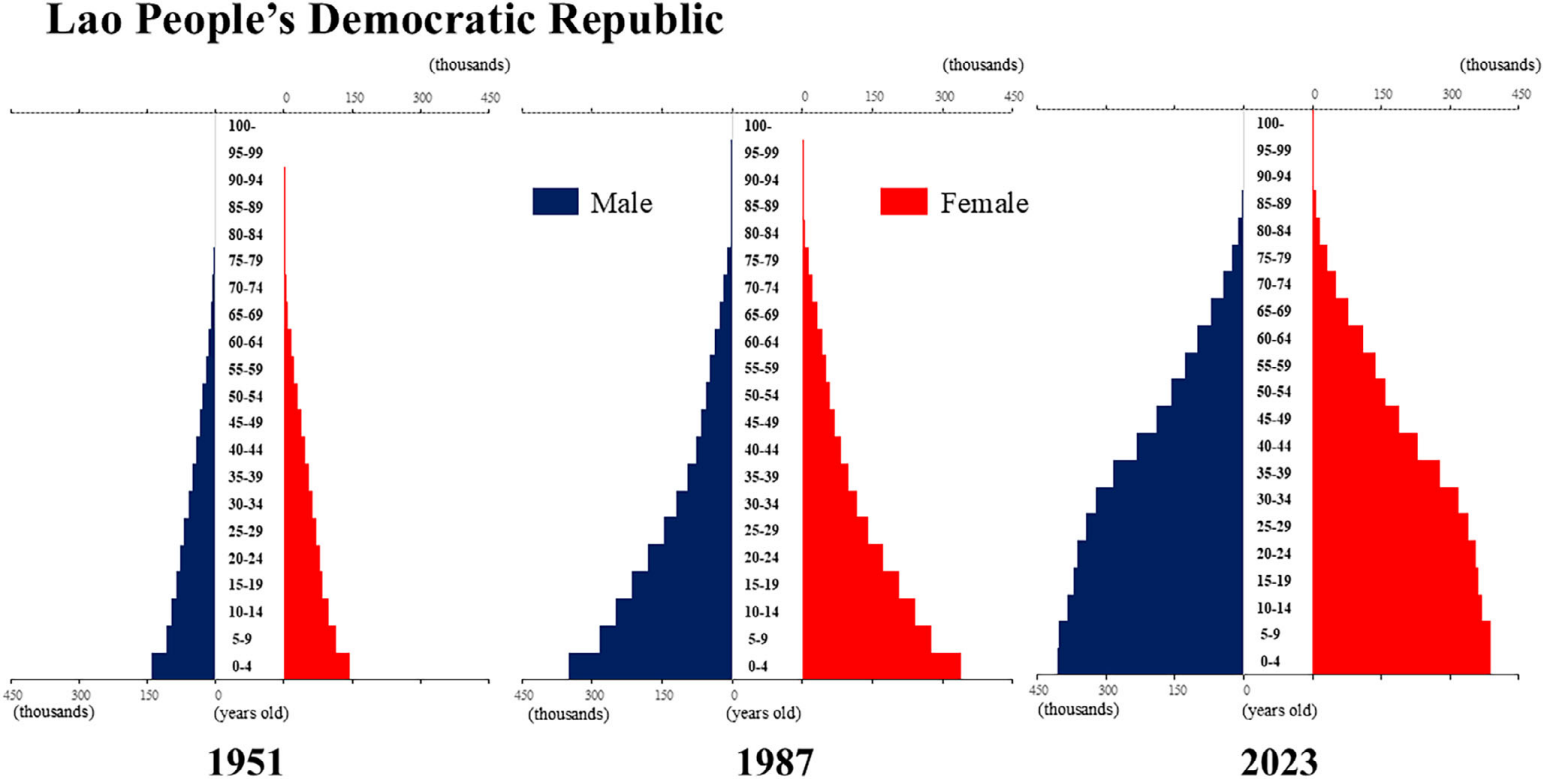
Population pyramid: Laos. Data from United Nations, Department of Economic and Social Affairs, Population Division (2024). World Population Prospects 2024, Online Edition.

**Figure 7:**
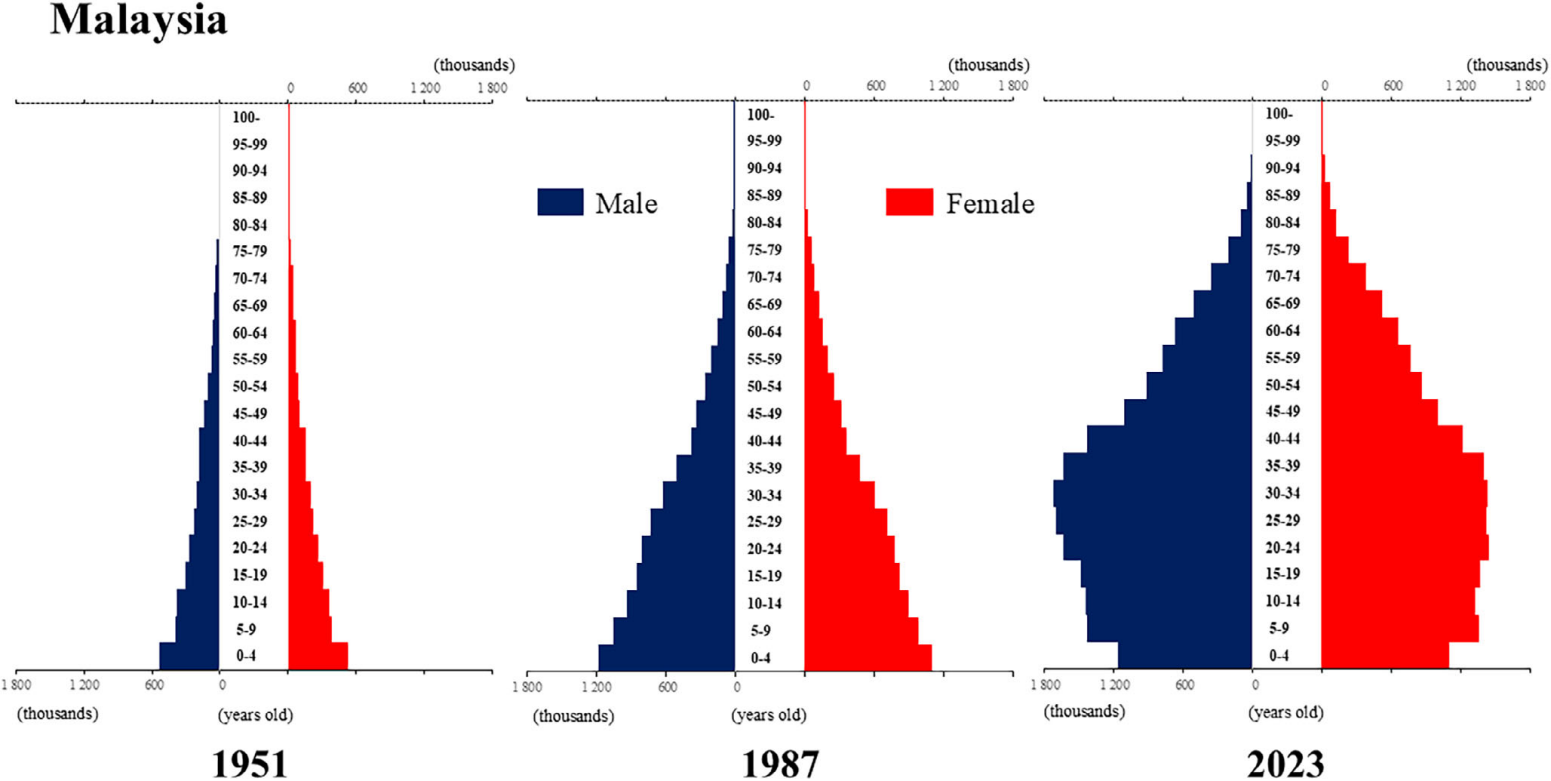
Population pyramid: Malaysia. Data from United Nations, Department of Economic and Social Affairs, Population Division (2024). World Population Prospects 2024, Online Edition.

**Figure 8:**
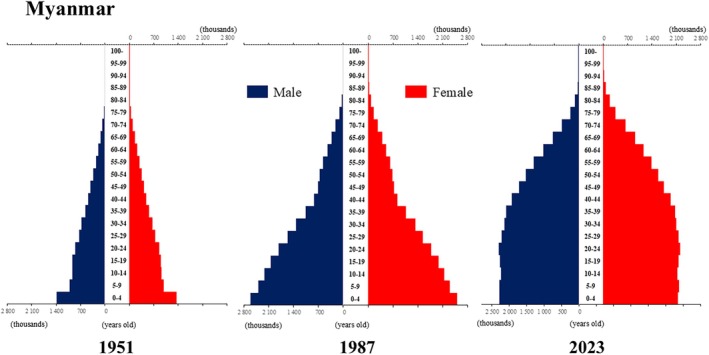
Population pyramid: Myanmar. Data from United Nations, Department of Economic and Social Affairs, Population Division (2024). World Population Prospects 2024, Online Edition.

**Figure 9:**
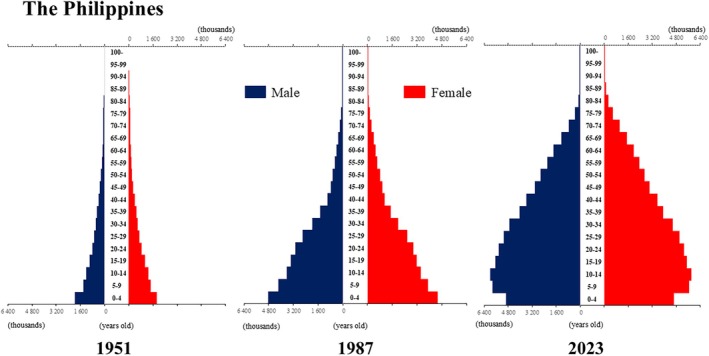
Population pyramid: the Philippines. Data from United Nations, Department of Economic and Social Affairs, Population Division (2024). World Population Prospects 2024, Online Edition.

**Figure 10:**
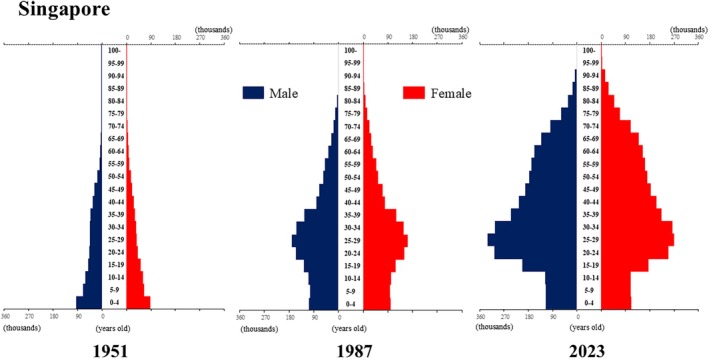
Population pyramid: Singapore. Data from United Nations, Department of Economic and Social Affairs, Population Division (2024). World Population Prospects 2024, Online Edition.

**Figure 11:**
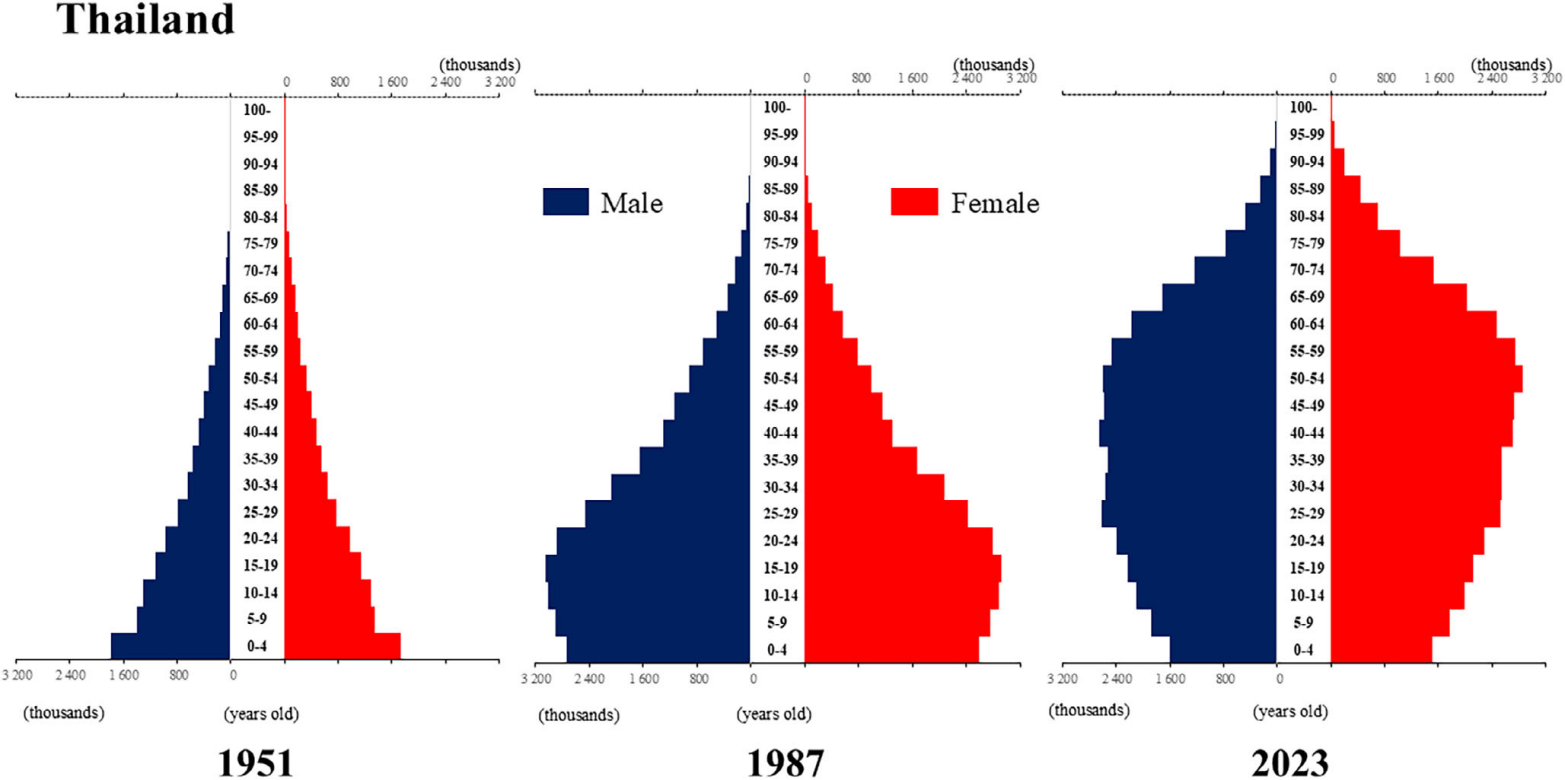
Population pyramid: Thailand. Data from United Nations, Department of Economic and Social Affairs, Population Division (2024). World Population Prospects 2024, Online Edition.

**Figure 12:**
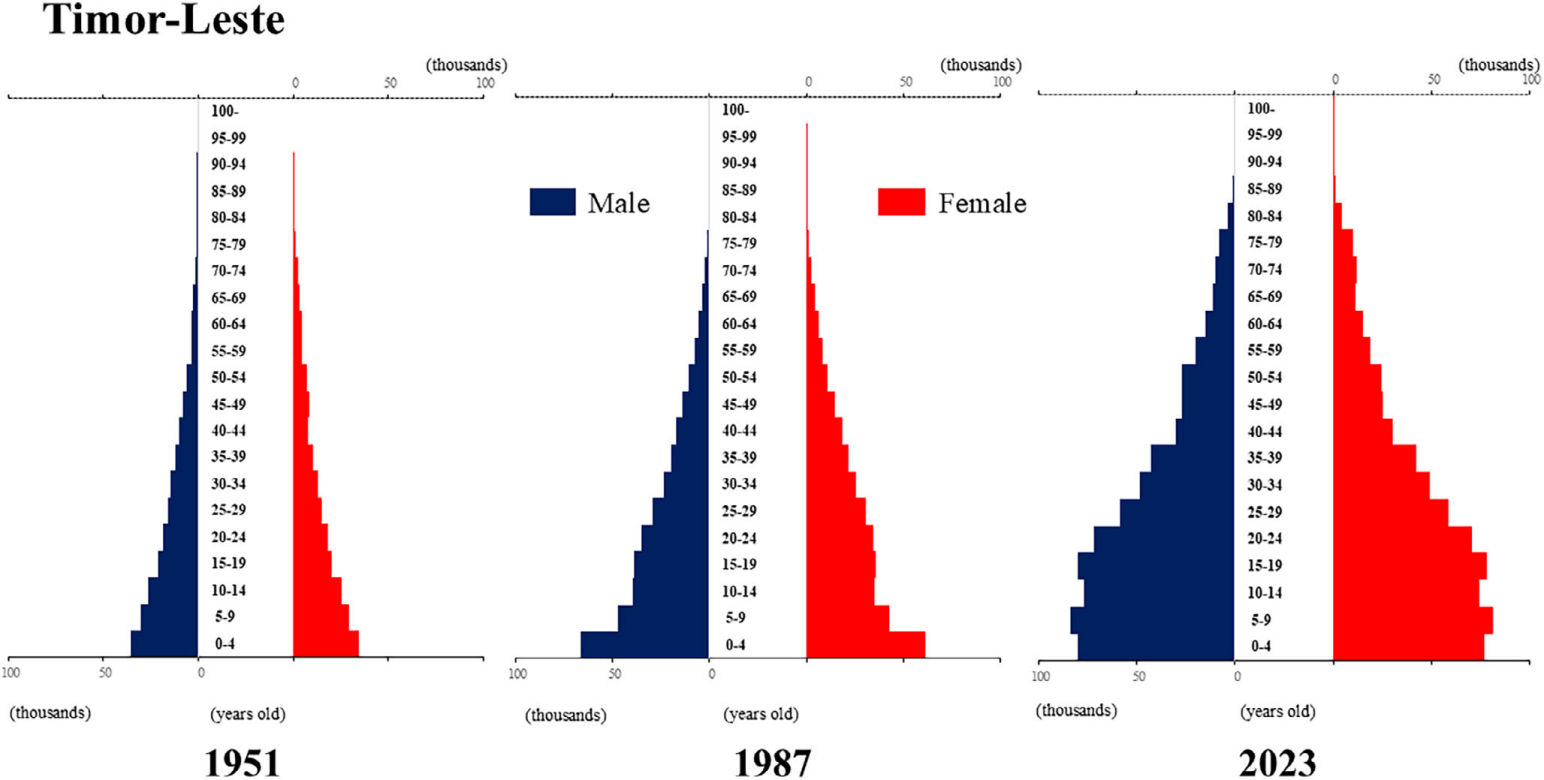
Population pyramid: Timor‐Leste. Data from United Nations, Department of Economic and Social Affairs, Population Division (2024). World Population Prospects 2024, Online Edition.

**Figure 13:**
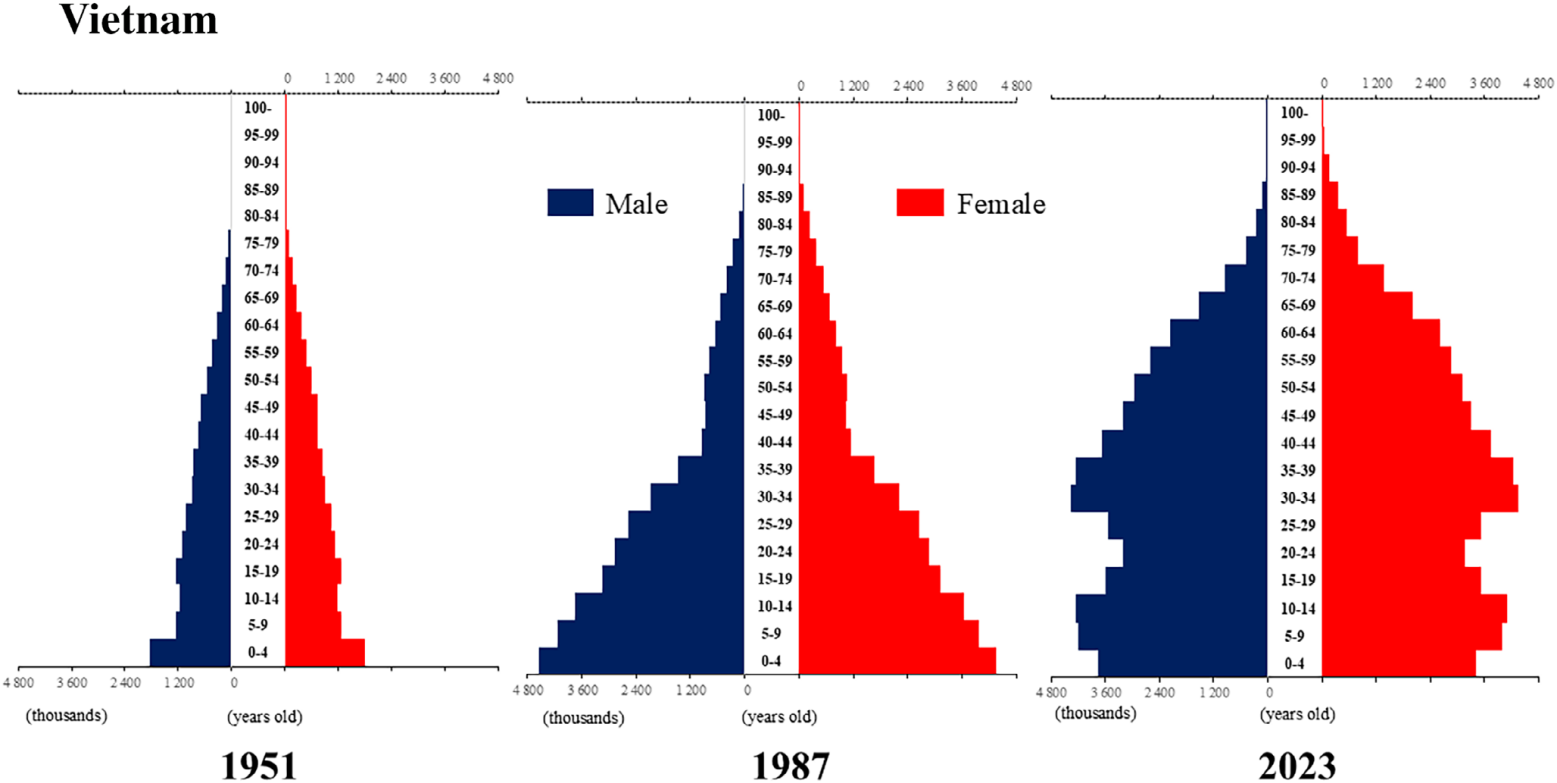
Population pyramid: Vietnam. Data from United Nations, Department of Economic and Social Affairs, Population Division (2024). World Population Prospects 2024, Online Edition.

**Figure 14:**
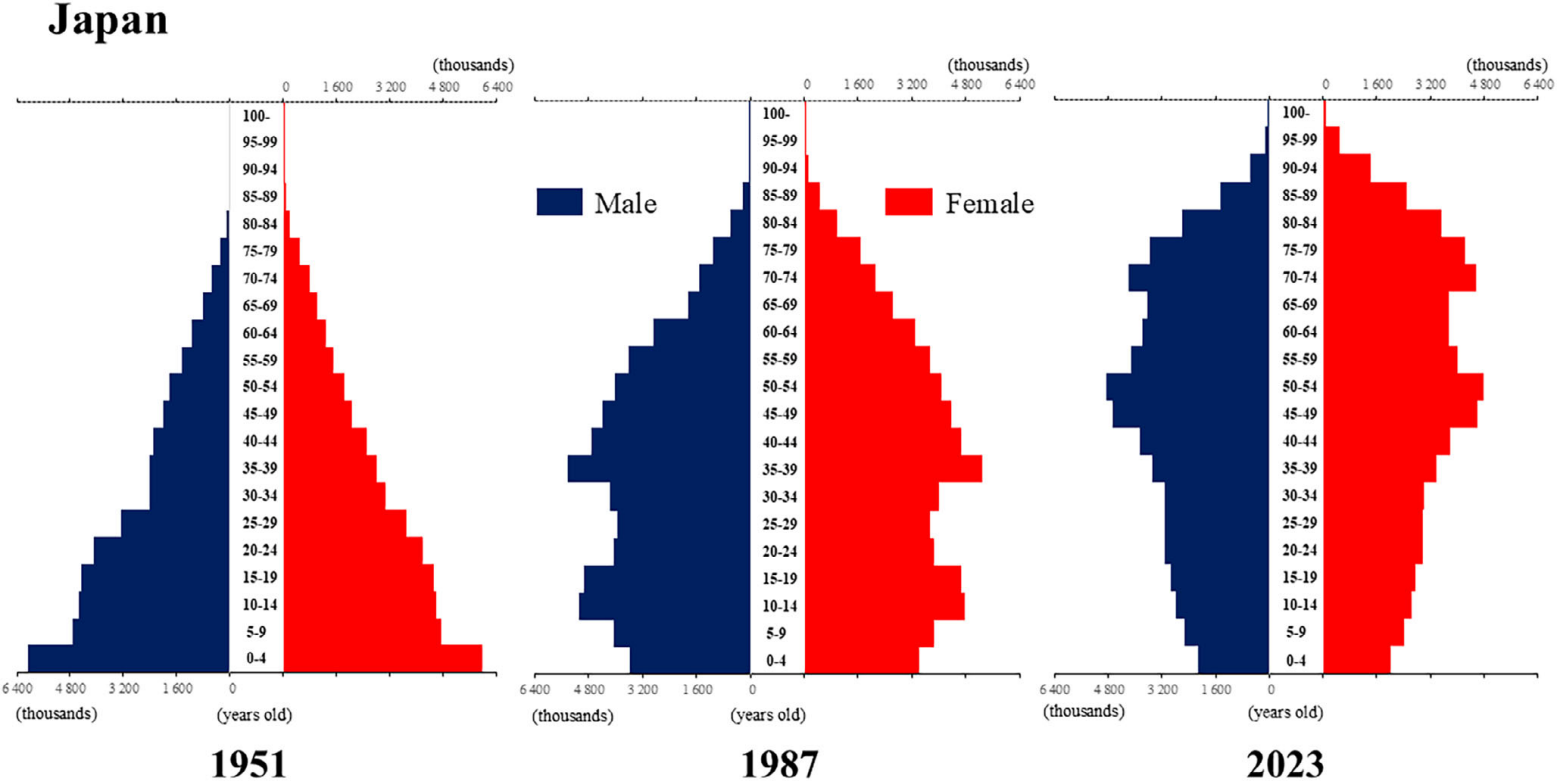
Population pyramid: Japan. Data from United Nations, Department of Economic and Social Affairs, Population Division (2024). World Population Prospects 2024, Online Edition.

**Figure 15:**
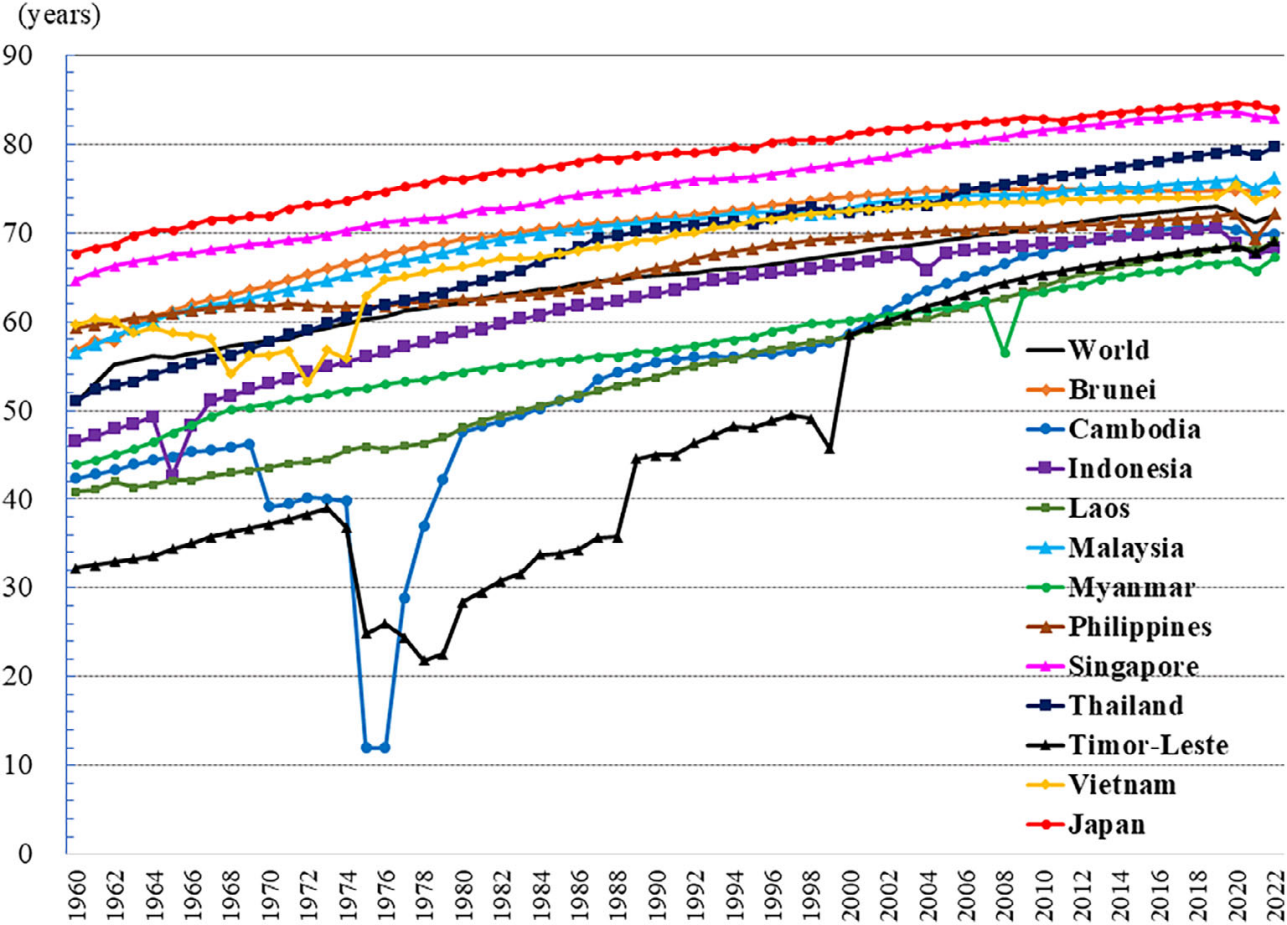
Trend in life expectancy at birth. Data from World Bank Group, DataBank, World Development Indicators. Last updated: 16 December 2024.

The global population is aging at an accelerating pace. The percentage of older adults aged >65 years, which was approximately 5% in 1950, began to increase rapidly around the 1990s, reaching 10% by 2023 (Fig. 1). This shift has resulted in a transformation of the population pyramid into a bell‐shaped structure (Figs 2, 3, 4, 5, 6, 7, 8, 9, 10, 11, 12, 13, 14).^1^ Furthermore, the global life expectancy at birth, which was approximately 48 years in 1950, surpassed 71 years by 2023 (Fig. 15).^2^ Humanity's longevity is the result of social development, including health systems, but it also poses challenges. Aging increases the risk of various diseases, such as cardiovascular disease (CVD), malignant neoplasms, chronic respiratory disease, dementia, diabetes mellitus (DM) and musculoskeletal disorders, all of which impose substantial medical costs and caregiving labors. As a result, there is growing discussion regarding the need for society to address the increasing challenges.^3^, ^4^

The aging of the population is generally observed in Southeast Asia, with accelerated aging particularly noticeable in Thailand and Singapore (Fig. 1, Table 1).^1^ Of the 11 countries in Southeast Asia, Thailand (14.7%) is classified as an “aged society” (defined as 14–21% of the population aged ≥65 years), whereas Singapore (13.1%), Vietnam (8.6%), Malaysia (7.5%), Myanmar (7.1%) and Indonesia (7.0%) are classified as “aging societies” (defined as 7–14% of the population aged ≥65 years). In particular, Japan (29.6%) is classified as a “super‐aged society” (defined as >21% of the population aged ≥65 years).

**Table 1:** Basic information of Southeast Asian countries and Japan

Indicator	Brunei	Cambodia	Indonesia	Laos	Malaysia	Myanmar	Philippines	Singapore	Thailand	Timor‐Leste	Vietnam	Japan
Political Regime	Absolute monarchy	Constitutional monarchy	Presidential republic	Communist state	Constitutional monarchy	Military regime	Presidential republic	Parliamentary republic	Constitutional monarchy	Semi‐presidential republic	Communist state	Constitutional monarchy
Population (thousands)*	459	17 424	281 190	7665	35 126	54 134	114 891	5789	71 702	1384	100 352	124 371
People aged ≥65 years (%)*	6.5	6.0	7.0	4.5	7.5	7.1	5.3	13.1	14.7	5.3	8.6	29.6
90th percentile age (years)*	60	59	61	56	61	61	58	68	69	55	63	80
Total fertility rate^†^	1.76	2.32	2.15	2.45	1.79	2.13	2.73	1.04	1.32	3.05	1.94	1.26
Life expectancy at birth (years)^‡^	74.6	69.9	68.3	69.0	76.3	67.3	72.2	82.9	79.7	69.1	74.6	84.0
Maternal mortality ratio per 100 000 live births^¶^	44.2	218.0	172.9	126.1	21.1	178.7	78.2	7.5	28.6	203.9	45.5	4.3
Neonatal mortality rate per 1000 live births^¶^	5.0	12.2	10.7	20.3	4.4	21.1	14.2	0.9	4.5	21.7	10.4	0.8
<5 years‐of‐age mortality rate per 1000 live births^¶^	9.7	23.7	21.3	40.4	7.8	40.1	27.5	2.2	8.1	48.6	20.3	2.3
Age‐adjusted prevalence of diabetes (%)^¶^	11.1	7.3	10.6	6.2	19.0	7.1	7.1	11.6	9.7	8.6	6.1	6.6
Obesity among adults (%)^¶^	31.7	4.4	11.2	8.0	22.1	7.4	8.7	13.9	15.4	2.4	2.0	5.5
Tobacco use (%)^¶^	16.4	17.2	38.2	27.2	22.0	44.4	20.4	16.4	19.2	38.7	22.5	19.2
Mortality attributed to air pollution (per 100 000 population)^¶^	19.6	163.3	96.1	195.3	76.4	184.1	202.8	23.4	46.5	185.6	102.8	11.8
TB incidence (per 100 000 population)^¶^	57	320	385	138	113	475	638	51	155	498	176	10
Road traffic mortality (per 100 000 population)^¶^	3.6	18.8	11.3	16.4	13.9	19.3	9.7	1.9	25.4	12.0	17.7	2.7
Suicide mortality rate (per 100 000 population)^¶^	2.7	4.7	1.2	4.6	5.6	2.9	3.6	8.4	16.4	3.6	7.6	17.5
Medical doctors per 10 000 population¶	19.1	2.1	6.9	3.3	23.2	7.5	7.9	26.0	9.3	7.7	9.8	26.1
Nursing and midwifery personnel per 10 000 population¶	67.1	10.2	41.7	11.8	33.9	11.0	47.5	61.8	30.8	17.7	14.5	124.5
UHC service coverage index^¶^	78.3	58.0	54.8	51.8	76.0	52.4	58.2	88.5	82.0	52.3	68.1	83.5
GDP per capita (USD)^||^	33 577	2546	4920	1971	12 091	1190	3906	84 734	7336	1761	4324	33 899
CHE per capita (USD)**	693	122	161	69	487	65	203	3970	364	135	173	4437
OOP (% of CHE)**	6.8	54.9	27.5	30.9	32.1	70.3	44.6	22.5	9.0	5.9	40.0	12.0

CHE, current health expenditure; GDP, gross domestic product; OOP, out‐of‐pocket expenditure; TB, tuberculosis; UHC, universal health coverage.

* Data from United Nations, Department of Economic and Social Affairs, Population Division (2024). World Population Prospects 2024, Online Edition.

† Data from World Bank Group, DataBank, Gender Statistics. Last Updated: 17 December 2024.

‡ Data from World Bank Group, DataBank, World Development Indicators. Last Updated: 16 December 2024.

¶ Data from World Health Statistic 2024, Latest available as of May 2024, by indicator and country, area, WHO region.

|| Data from International Monetary Fund, World Economic Outlook Database, October 2024.

** Data from World Health Organization, The Global Health Observatory; United Nations, Department of Economic and Social Affairs, Population Division (2024). World Population Prospects 2024, Online Edition.

## Life expectancy, historical calamities and their impact on older adults

Life expectancy has been increasing worldwide (Fig. 15). However, in 2019 and 2021, a global decline in life approximately was observed. It is estimated that about 14.9 million excess mortality due to COVID‐19 occurred worldwide during the 2‐year period.^5^ The severity of COVID‐19 was strongly influenced by age, with the risk of death being found to be 23‐fold higher in individuals aged >65 years compared with younger populations.^6^

In Southeast Asia, advances in maternal and child health have contributed to a reduction in child mortality.^7^ However, neonatal deaths remain a serious problem, especially in Timor‐Leste, Myanmar and Laos (Table 1). Significant disparities are evident not only between countries, but also within countries. For example, in Laos, the mortality rate of children aged <5 years was reported to be significantly higher among the minority groups: Mon‐Mien (44 per 1000), Mon‐Khmer (63 per 1000) and Chinese/Tibetan (72 per 1000) compared with the majority Lao/Thai (35 per 1000), in 2017.^8^ In Cambodia, the mortality rate of children aged <5 years for the period 2021–2022 in the combined provinces of Mondulkiri and Ratanakiri was more than eightfold higher than in Phnom Penh, with rates of 42.3 per 1000 live births and 5.2 per 1000 live births, respectively.^9^ In Myanmar, the 2023 government report showed that life expectancy at birth was lower in Chin (63.9 years), Kayin (64.4 years) and Mon (64.6 years) compared with Nay Pyi Taw (69.5 years).^10^ In Indonesia, the life expectancy at birth in the top three provinces, Bali, North Kalimantan and Jakarta, is 74 years, whereas the lowest three provinces of Papua – North Maluku and West Sulawesi – is 66 years, a difference of 8 years in life expectancy.^11^

The trend in life expectancy shows significant declines in certain periods of several countries (Fig. 15). The Cold War has partially affected these declines.^12^, ^13^ In Vietnam, starting in the 1950s, the US aimed to prevent the spread of communism, influenced by the domino theory. In 1964, the Gulf of Tonkin incident, in which the US destroyer, Maddox, was attacked by North Vietnamese forces (later found to contain false information), led to all‐out war.^14^ In 1965, the US initiated full‐scale bombing of North Vietnam and deployed ground troops to South Vietnam, escalating the conflict into a full‐scale war. Vietnam's patient guerrilla warfare tactics, coupled with the growth of a broad anti‐Vietnam War movement both domestically and internationally, ultimately led to the withdrawal of the US troops by 1973. A study from Vietnam showed that exposure to wartime stressors was associated with the risk of post‐traumatic stress disorder and CVDs, and that severe childhood hunger and environmental hardships were linked to cognitive decline in later life.^15^

As the Vietnam War intensified, pro‐US Marshal Lon Nol staged a coup d'état in Cambodia in 1970 against the Sihanouk regime, which had adopted an anti‐US stance. Thereafter, the US invaded Cambodia to disrupt the supply routes between North Vietnam and the National Front for the Liberation of South Vietnam. As a consequence, civil war erupted between the pro‐US Lon Nol government forces, Sihanouk's former government forces and the Khmer Rouge. Heuveline estimated that, during the period 1970–1979, excess deaths ranged from 1.17 million to 3.42 million.^16^ Importantly, this estimate excludes deaths of children born during this period from the total of excess deaths.^16^ A large number of men, particularly those from urban areas and with higher education, died as a result of violence. Infant mortality was also very high during this period.^17^ The Khmer Rouge regime is noted to have left long‐lasting effects not only on poverty and healthcare, but also on family support structures for older adults.^18^, ^19^, ^20^

In Timor‐Leste, conflict occurred between 1974 and 1999. Silva *et al*. estimated that at least 102 800 individuals lost their lives during this period, with 18 600 fatalities attributed to conflict‐related violence, and 84 200 deaths resulting from hunger and disease.^21^, ^22^ Hunger claimed many victims, especially children and older adults.^23^ After Portugal's decision to withdraw from its colony after the Carnation Revolution in Lisbon in 1974, a civil war broke out between the Revolutionary Front for an Independent East Timor (FRETILIN), the Timorese Democratic Union and the Timorese Popular Democratic Association. Taking advantage of the country's division, Indonesian troops invaded under the pretext of preventing instability and the spread of communism in neighboring areas. This invasion resulted in nearly 25 years of annexation and brutal military occupation.^24^ Meanwhile, the FRETILIN fled to the mountains and continued their resistance movement. Many residents were forcibly relocated not only within the country, but also to Indonesia, leaving a lasting impact on the care of older adults in the form of fragmentation of relatives after the conflict.^25^

In May 2008, Cyclone Nargis struck the Irrawaddy Delta of southern Myanmar, sweeping villages away with a tidal surge. With winds exceeding 200 km/h and a 3.6‐m high wall of water, tens of thousands died, and hundreds of thousands lost their homes, and many were left in vulnerable conditions, suffering from injuries and illness.^26^ HelpAge International reported that many older adults were still complaining that their lives had not recovered and that their health situation had deteriorated, even 9 months after the cyclone.^27^

In Indonesia, there were clear declines in life expectancy from 1965 to 1966 and in 2004. During that period, the Indonesian military and associated civilian militia groups eliminated the leadership of the Indonesian Communist Party along with numerous party members. Estimates of the death toll range from 100 000 to 2 million.^28^, ^29^, ^30^ On 26 December 2004, a moment magnitude 9.1–9.3 earthquake struck off the west coast of Aceh Province in northern Sumatra. After the earthquake, a tsunami with a run‐up of up to 50.9 m was observed in Aceh Province. The tsunami is reported to have caused 227 899 deaths or missing persons across 15 countries, and displaced 1.7 million people. Indonesia was the hardest‐hit country, with 167 540 people reported dead or missing.^31^ According to a report from Aceh, the highest mortality rates from the tsunami were observed among adults aged >70 years.^32^

The coup d'état and subsequent violence in Myanmar, which commenced on 1 February 2021, caused significant disruption to healthcare, with shortages of medical staff and medicines. This severely compromised the quality of medical services, further exacerbating the damage caused by the COVID‐19 pandemic. The healthcare infrastructure was severely degraded. Healthcare workers faced ongoing insecurity due to bombings and arrests, with many fleeing to rural areas, working clandestinely, and experiencing heightened psychological distress and strain as a result of resistance movements and protests against the regime.^33^, ^34^ It has been reported that, as of 30 June 2024, since 1 February 2021, at least 5350 civilians have been killed, >3 million individuals have been displaced and >15 million are food insecure.^35^ It was reported that medical units were also destroyed and occupied by military forces, and at least 880 health workers have been arrested, 97 killed and 117 injured.^36^

## Falling fertility rate

**Figure 16:**
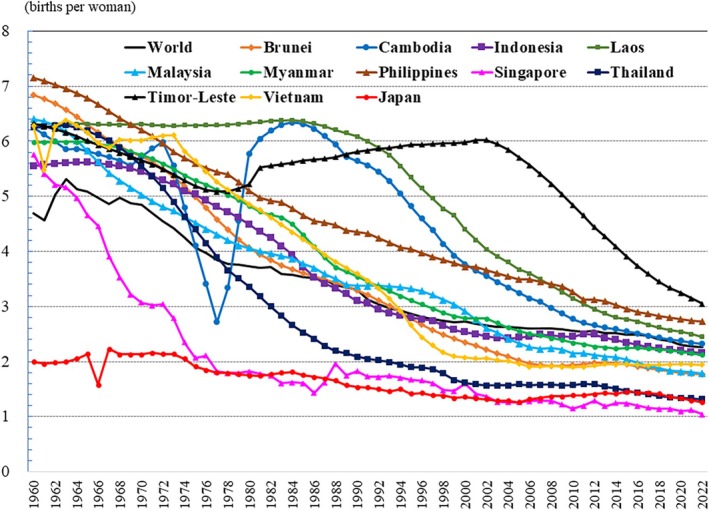
Trend in total fertility rate. Data from World Bank Group, DataBank, Gender Statistics. Last updated: 17 December 2024.

The trends in fertility rates over time suggest that the effects of the aforementioned calamities in Cambodia and other countries are also evident in fertility rates (Fig. 16). Apart from that, it is observed that fertility rates are declining overall in Southeast Asia (Fig. 16). Five (Singapore, Thailand, Malaysia, Brunei and Vietnam) of the 11 countries in Southeast Asia have fertility rates under the replacement level of 2.1.^1^ In particular, the fertility rates are significantly low in Singapore (1.04) and Thailand (1.32), as it is in Japan (1.26; Table 1).^1^

Various factors have been pointed out as contributing to the declining fertility, including family planning, which was activated in the 1970s, a decrease in the number of children desired, increase in singlehood and later marriages, and lack of support, such as maternity leave and daycare.^37^, ^38^, ^39^, ^40^ Interesting points in the reports from Thailand are that culturally, women are more likely to bear the economic burden for caring their parents, and that the fertility is more preserved in the southern region, where there are more Muslims, than in other regions.^37^, ^38^ It has been reported that some Muslims in southern Thailand tend to view contraception as *haram* (forbidden).^41^ Since the 1980s, Singapore has eased its immigration policy by implementing a strategy to attract skilled foreign workers and talented international students by their standards, encouraging them to apply for permanent residency and citizenship, whereas offering only short‐term permits to others.^39^, ^42^ In 1987, Singapore implemented a population policy encouraging families to have three children, or more if they can afford it.^43^ The government introduced incentives including tax rebates, childcare subsidies and the use of medical savings to cover delivery costs for the first three children. Additionally, parents who had a third child were given priority in the allocation of larger housing. The fertility rate increased in 1988, but it subsequently declined. The tentative increase in 1988 is believed to have been influenced more by cultural beliefs regarding the auspiciousness of births during the Year of the Dragon.^39^, ^40^ In 2001, Singapore introduced a baby bonus scheme to increase the birth rate. So far, the effects have not been dramatic, but the system is being amended to increase benefits in 2023, and future results will be closely watched.^44^

The tentative fertility decline in Japan in 1966 is attributed to the superstitious belief of the “*hinoe‐uma*” (fire‐horse), which occurs once every 60 years in the oriental zodiac.^45^ In Japan, the fertility rate has remained <2.1 since the mid‐1970s, but it was not until 1990, when the fertility rate fell to 1.57, that the government started to seriously consider how to address the issue. Since 1994, a lump‐sum allowance for childbirth and childcare has been provided, and various policies have been implemented to improve the birth and childcare environment, including the introduction of parental leave, the expansion of nursery schools, free preschool education and childcare, subsidized medical care for children aged <18 years, child support allowances, and schooling support. However, the fertility rate has not improved. Japan's child population continues to decline, and the shape of the country's population pyramid is now approaching that of a torch (Fig. 14).

## Diseases and modifiable risk factors

Based on the World Health Organization database, CVDs, such as ischemic heart disease (IHD) and stroke, were one of the leading causes of deaths in all Southeast Asian countries and Japan, with the aging population.^5^ Reducing risk factors for CVDs, such as DM, obesity, tobacco, hypertension and dyslipidemia, is one of the important global issues.

The prevalence of DM is particularly high in Malaysia, obesity rates are elevated in Brunei, as well as Malaysia. Studies in Brunei and Malaysia indicate that contributing factors to DM and obesity include sedentary lifestyles, increased sugar consumption, frequent snacking and consumption of fried foods from adolescence, breakfast skipping, and low intake of vegetables and fruits.^46^, ^47^, ^48^, ^49^, ^50^, ^51^ In Malaysia, the risk is higher among Malay and Indian populations compared with the Chinese.^51^ Notably, the indigenous Orang Asli population faces a dual burden of malnutrition, with both undernourished individuals showing anemia, wasting, stunting and micronutrient deficiencies, and overnourished individuals presenting with obesity and diabetes.^52^ Additionally, a high incidence of neglected tropical diseases, including soil‐transmitted helminth infections and amoebiasis, has been reported among the Orang Asli.^52^

Tobacco use is widespread in Myanmar, where both cigarettes and cheroots are commonly consumed (Table 1). People also hand‐roll tobacco using bark, stems, roots and various leaves. Notably, many individuals begin tobacco use before the age of 14 years.^53^ A report from the Ayeyawady region indicates that residents lack awareness of the risks of passive smoking, even in the presence of pregnant women.^54^ The regions of Mandalay and Magway are known to be the largest tobacco‐producing areas.^55^ In some cases, individuals consume raw and cured tobacco leaves mixed with alcohol, honey, lime and other substances.^56^ Betel quid chewing, known locally as “*kunya*,” is prevalent not only among men, but also among women.^57^ This practice involves wrapping the drupe of *Areca catechu* in the leaf of *Piper betel* smeared with slaked lime, sometimes combined with tobacco leaves. Offering and receiving *kunya* while engaging in conversation is a custom in Myanmar.

Air pollution is an important modifiable risk factor and is associated with a range of health problems, including ischemic heart disease, stroke, lower respiratory tract infections, chronic obstructive pulmonary disease and lung cancer, due to cumulative exposure from childhood, as well as acute symptoms.^58^ In Southeast Asia, air pollution results from various sources. In the Philippines, average PM2.5 levels at traffic sites in Metro Manila during the dry season reached 58.4 μg/m^3^, with vehicular emissions identified as the primary source.^59^, ^60^ In Timor‐Leste, rural households rely on wood burning for cooking, creating significant indoor air pollution.^61^ In Laos, vegetation fires, partly from shifting cultivation, contribute to most PM2.5 pollution, with industrial emissions, waste incineration and road traffic being major contributors in urban areas, such as Vientiane.^62^ In rural areas, such as Savannakhet province, residential cooking and crop residue burning are key sources.^62^ In Indonesia, peatland fires in 2019 were reported to have caused >3200 excess deaths in Central Kalimantan, and >87 000 excess deaths nationwide due to PM2.5 exposure.^63^

Tuberculosis is still a significant cause of death (Table 1). In the Philippines, an estimated 7.6 million people live in urban slums, and the risk of contracting tuberculosis is particularly high among the urban poor, HIV‐infected people, people living with diabetes, health workers, smokers and older adults.^64^ Although the incidence of cancer is expected to rise in other Southeast Asian countries, a critical issue remains the establishment of a system that ensures equitable access to proper diagnosis and effective treatments for patients across diverse regions.

In Thailand, the number of reported road traffic deaths reached 16 957 in 2021.^65^ Regulations regarding drunk driving, as well as the use of seatbelts and helmets, are established; however, compliance with these regulations remains inadequate. According to 2021 survey in Thailand, only half of individuals with a drinking habit reported refraining from drinking and driving, and only 7.4% of riders wore helmets safely among motorcycle fatalities.^65^ A survey in Thailand between 2010 and 2011 reported that the seat belt usage rate among motor vehicle drivers was 41.2%.^66^ Statistics show that both the aging population and older adults are at an elevated risk of traffic accidents, as drivers and pedestrians, highlighting the urgent need for effective countermeasures.^67^

## Health coverage and barriers

The universal health coverage service coverage index is calculated based on 14 indicators that assess service capacity and access to maternal and child healthcare, as well as care for both communicable and non‐communicable diseases (Table 1).^68^ This index is relatively low in Laos (51.8), Timor‐Leste (52.3) and Myanmar (52.4; Table 1).

As of 2023, the public medical system in Laos had five central hospitals, 17 provincial hospitals, 135 district hospitals and >1074 health centres.^69^ Private medical facilities in Laos are limited and are usually simultaneously staffed by medical personnel from public medical facilities. In Laos, service delivery remains a challenge due to limited geographic access to health facilities, particularly in regions with forested mountains and steep terrain, especially during the rainy season.^70^ A survey carried out in the Bokeo, Xaisomboun, Khammouane and Champassak provinces of Laos showed that patients' trust in health centers might be undermined by non‐responsiveness to emergencies, unjust financial demands, favoritism toward relatives, and discrimination against minorities and economically disadvantaged populations.^71^

In Timor‐Leste, it has been reported that the public health system consists of one national referral hospital located in the capital city of Dili, five regional referral hospitals, 75 community health centers and 442 health posts.^72^ However, access to medical care has become a significant challenge.^73^ Despite public health services being free, the effort and cost of traveling from rural and mountainous areas to hospitals have created substantial barriers to healthcare access. It has been reported that one in four households is >2 h walk from the nearest health facility.^74^ *Serviço Integrado Saúde Communitária* and *Saúde na Familia* take an important role of visiting community in each village to identify diseases. Although the government provides a monthly stipend of $30 to people aged ≥60 years, this amount is insufficient to cover living expenses, and specialized care facilities for older adults are lacking. However, the culture of being cared for by family and relatives has taken root.^75^ It has been reported in Timor‐Leste that folk remedies and healing (*humere*) methods using bark, fruit, leaves, sap, flowers and roots are widely spread, and some healers (*navarana* or *inaharan*) use methods such as dream analysis.^74^, ^76^, ^77^

There is a significant shortage of medical professionals, particularly in Cambodia (Table 1). Under the Khmer Rouge regime, doctors were classified as “intellectuals” (*panh nha chun*) and were largely considered enemies.^78^ Most doctors were massacred, and died from disease, exhaustion and starvation, or fled the country.^79^ It has been noted that the effects of the elimination of medical professionals and the closure of University of Health Sciences, Cambodia's only medical school at that time, under the Khmer Rouge regime still remain.^79^ Currently in Cambodia, there are 34 national and provincial (*khaet*) / municipal (*krong*) hospitals, 92 Operational District referral hospitals, 1222 health centers (HCs) and 128 health posts.^80^ Health centers provide preventive services, basic curative care and delivery services, and oversee health posts. Each HC covers a population between 10 000 and 20 000. The health Operational Districts typically supervise 10–15 HCs, covering a population of approximately 100 000 to 200 000 people. Health posts are to be located at least 15 km from the nearest HC, often in areas with geographical obstacles such as rivers, mountains and poor roads. In addition to so‐called modern medical practitioners, Cambodia is home to various indigenous healers known as *kru khmae*, who utilize medicinal herbs, perform bone‐setting procedures, and conduct rituals, mantras, divination, mediumship and so on.^78^ The Bunong people, primarily residing in Mondulkiri, are reported to consider themselves part of an environment they share with spirits and ancestors.^81^ Some believe spirit‐gods (*brah‐yaang*) are present in mountains, forests, rivers and so on, and influence their health and well‐being.^82^ Healers, referred to as *bu blao* in the Bunong language, are reported to use a variety of natural resources, including plants, mushrooms, mammals, birds, reptiles, insects and minerals, and perform rituals and engage with spirits through these practices to diagnose and treat illnesses.^83^

An important indicator of health coverage is the share of out‐of‐pocket (OOP) costs in current health expenditure, which reflects the extent to which health costs are directly financed by households (Table 1). High out‐of‐pocket payments are linked to catastrophic health expenditure, typically defined as OOP >10% or 25% of total household consumption or income, or >40% of non‐food consumption.^84^, ^85^ It has been noted that households with older adults or disabled persons might have a risk for catastrophic health expenditure.^86^ In Myanmar, since 2021, a noticeable decrease in public health spending has been observed, now standing at 2.5% of the union budget in 2023/2024, as compared with 4.6% in 2018/2019, resulting in very high OOP and increased rates of medical impoverishment.^87^ In Myanmar, the role played by non‐state actors (NSAs), including civil society organizations and ethnic health organizations, has historically been very important, especially when considering health services in border areas.^88^ Since the military coup, NSAs have become even more significant, although they face substantial challenges in procuring essential medications and securing financial resources. Furthermore, substantial concerns have emerged regarding the delivery of emergency medical care, not only in the border areas where NSAs have a longstanding presence, but also in inner conflict zones, where the experiences of NSAs are relatively limited.^88^

In Southeast Asia, COVID‐19 was particularly devastating in Indonesia and the Philippines, with an estimated 1 million excess deaths in Indonesia and 230 000 in the Philippines.^89^ Both Indonesia and the Philippines are archipelagic countries. Delivering vaccines to remote areas often necessitates the use of various transportation infrastructures to maintain the cold chain, and fostering trust between the community and government is also a critical consideration. Additionally, the presence of pig‐derived and non‐halal ingredients in vaccines, along with the timing of Ramadan, are factors linked to vaccine hesitancy.^90^, ^91^

In Indonesia, in addition to 3095 hospitals, *Puskesmas* and *Posyandus* serve as essential centers of primary healthcare, with a projected total of 10 134 *Puskesmas* and 296 777 *Posyandus* nationwide by 2021.^92^ These facilities play a critical role in delivering organized services and act as the primary points of contact with the local population. Most *Puskesmas* have at least one doctor. *Posyandus* are health posts that function as outreach services of *Puskesmas*, staffed by community health workers, predominantly women, who are selected by and from the community. However, securing budget, equipment, and support from community leaders and *Puskesmas* presents a significant challenge for community health workers.^93^

In the Philippines, medical services are basically distributed at three levels: province, city/municipality and *Barangay*. As of the 2018 data, in addition to 1198 hospitals, there were up to 2593 basic medical facilities, such as rural health units, HCs and private medical clinics.^94^ Only half the population has access to the basic medical facility within a 30‐min radius of their residence. It has been pointed out that the situations were particularly severe in Bangsamoro, Bicol and Southwestern Tagalog.^94^ The government is working to establish at least one health station (HS) in each *Barangay*; however, as of the 2019 data, there were 22 613 HSs nationwide, with only half of the *Barangays* having access to at least one HS^94^. The majority of HSs in rural areas lack medical personnel. Overseas migration is also a key factor contributing to the shortage of medical staff, with an estimated one in five healthcare professionals working abroad.

In Thailand, during the spread of COVID‐19, >1 million village health volunteers nationwide supported local HCs by carrying out health education activities, disease surveillance and patient referrals.^95^ The pandemic has accelerated the adoption of digital health, such as telemedicine.^96^ It might be useful for older adults with multiple chronic diseases that require continuous monitoring, but have difficulty visiting health facilities. Digital health can also transcend geographical barriers, even national borders.^97^ In contrast, a study from Singapore showed that a significant proportion of older adults exhibited a lack of acceptance, desire and adherence toward telemedicine.^98^ The great challenge for our societies is to develop a healthcare system where no one is left behind, and everyone can receive medical care safely and securely.

## Future prospects

Considering the challenges of aging in Southeast Asian countries and Japan, although there are similarities in the increasing non‐communicable diseases as a proportion of deaths and the growing importance of digital health, including artificial intelligence, the current extent of aging and the quality of healthcare systems to support older adults vary significantly.

In addition, Southeast Asia is often hit by natural disasters, and is also a hotspot for emerging infectious diseases.^99^, ^100^ Yusuf and Francisco's analysis identifies high‐risk areas for climate‐related hazards, including drought‐prone regions in northwestern Vietnam, eastern Vietnam, southern Thailand, the Philippines, Sabah (Malaysia) and Java (Indonesia).^101^ Sea level rise and flood risks impact the Mekong River Delta (Vietnam/Cambodia), Bangkok and southern Thailand, Java (Indonesia), and central Luzon and southern Mindanao (the Philippines).^101^ Garner *et al*. estimated that tropical cyclones will increase in both peak intensity and duration in Hai Phong, Yangon and Bangkok.^102^ Taniushkina *et al*. projected that, by 2028, cropland area in Indonesia, Malaysia, the Philippines and Vietnam could decline by >10%, with rice production in Vietnam expected to decrease by 19% and in Thailand by 7%, compared with 2021 levels.^103^ Additionally, it has been projected that both the frequency and intensity of heatwaves will increase, and the distribution of vector‐borne diseases will change in Southeast Asia.^104^, ^105^ Southeast Asia is regarded as one of the regions severely affected by economic losses due to climate change. It is said that climate change might also exacerbate social tensions and strives over resources, undermine political stability and create a vicious circle that increases sovereign risk.^106^ Strengthening public finance resilience and mitigating sovereign risk through international cooperation are also critical challenges that need to be addressed. Although older people should not be uniformly fixed, it has been noted that, in general, older people might be more vulnerable to climate change impacts than other age groups.^107^

Especially regarding aging, it has been estimated that, if current trends persist, by 2088, the proportion of individuals aged ≥65 years in all Southeast Asian countries will exceed 21%, and the region will enter a “super‐aged society” similar to that of Japan today.^1^ As populations age, the prevalence of various chronic diseases is increasing. Of particular concern is the projected rise in the number of individuals with dementia, which is estimated to exceed 10 million in Southeast Asia by 2050.^108^ One of the essential elements for diagnosing major neurocognitive disorder is determining whether cognitive deficits interfere with an individual's independence in daily activities. Along with prevention and the development of treatments for cognitive decline, an essential aspect of the effort is to set up an environment that is friendly to individuals with cognitive decline. A friendly environment for people with cognitive decline should not necessarily be a highly developed society.^109^

**Figure 17:**
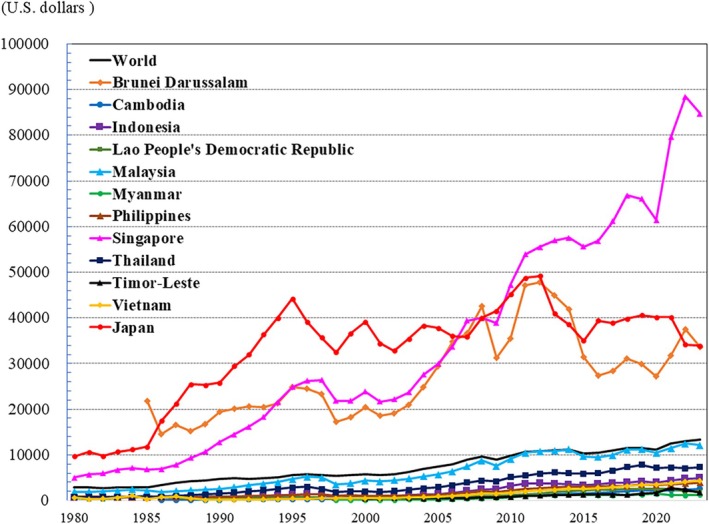
Trend in gross domestic product per capita, current prices. Data from International Monetary Fund, World Economic Outlook Database, October 2024.

As aforementioned, the proportion of the world's population aged ≥65 years currently stands at 10% (Fig. 1). In Japan, in contrast, the population aged ≥65 years has reached approximately 30% (Fig. 1). In Japan, employers are legally required to ensure employment until the age of 65 years, and to make efforts to employ until age 70 years. However, is this measure sufficient to get out of the “getting poor after getting super‐old” situation? (Fig. 17)^110^, ^111^ In Japan, it is estimated that by 2100, approximately 38% of the population will be aged ≥65 years, and 32% will be aged ≥70 years. Addressing the declining fertility rate is critically important. Immigration is another option to be considered. However, we must take into account the situation in neighboring countries, where the population is also aging. Our society has to confront the question of how society can manage with this age structure without turning our eyes away from it.

**Figure 18:**
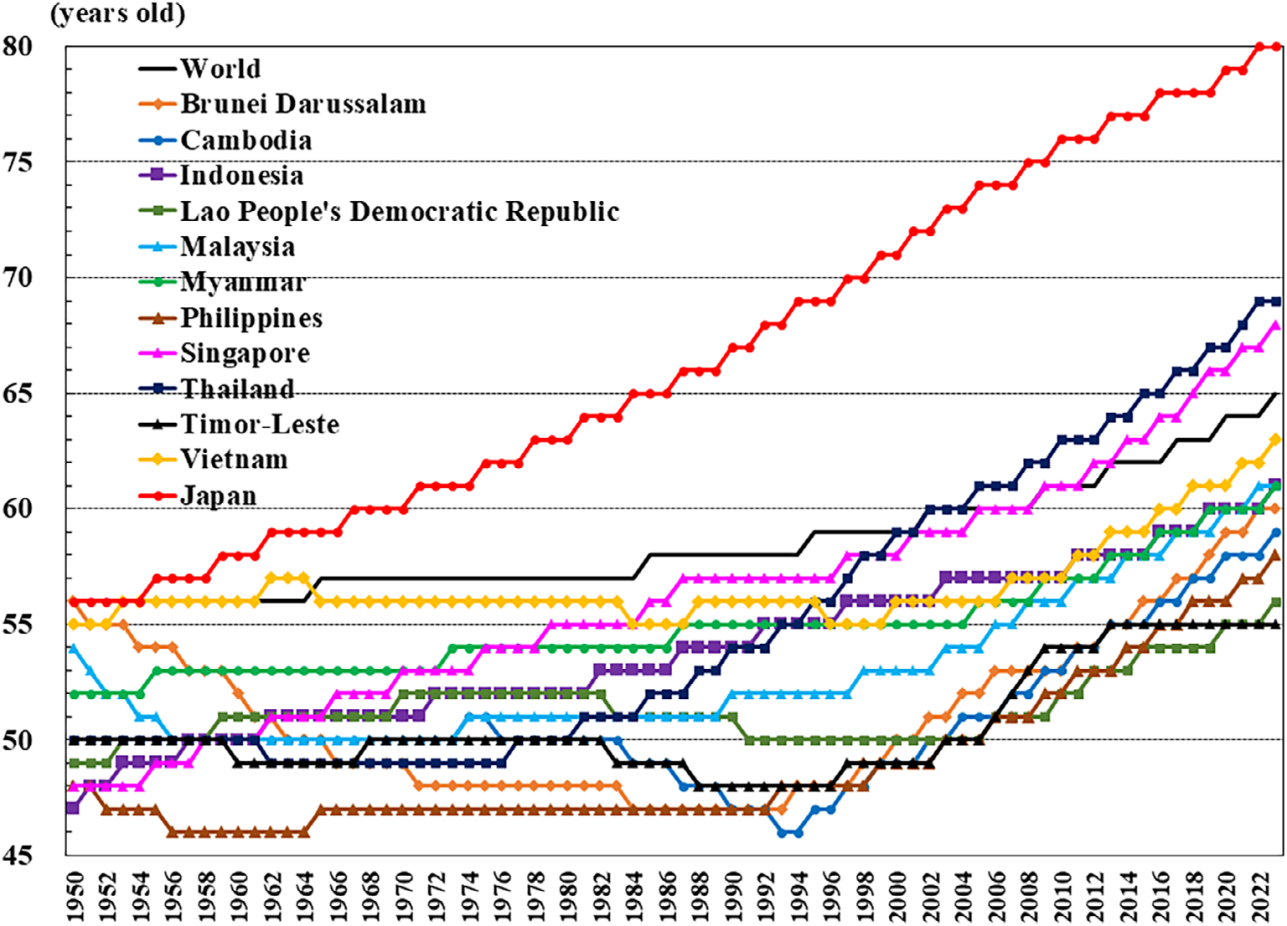
Trend in the 90th percentile of age. Data from United Nations, Department of Economic and Social Affairs, Population Division (2024). World Population Prospects 2024, Online Edition.

This paper focuses on the 90th percentile age over time, and advocates the importance of considering the relative position of age in society, rather than simply focusing on age alone. In the 1980s, the 90th percentile age in Japan was 65 years, which means that 10% of the population is aged ≥65 years (Fig. 18) However, the 90th percentile age in Japan has reached 80 years. The policy Japan could take is to create a society in which people can work even in old age, improving healthy life expectancy further and taking advantage of the high healthy life expectancy. It is important to have a flexible working system that accommodates the needs and beliefs of old and young men and women, with adaptable work locations and hours.

**Figure 19:**
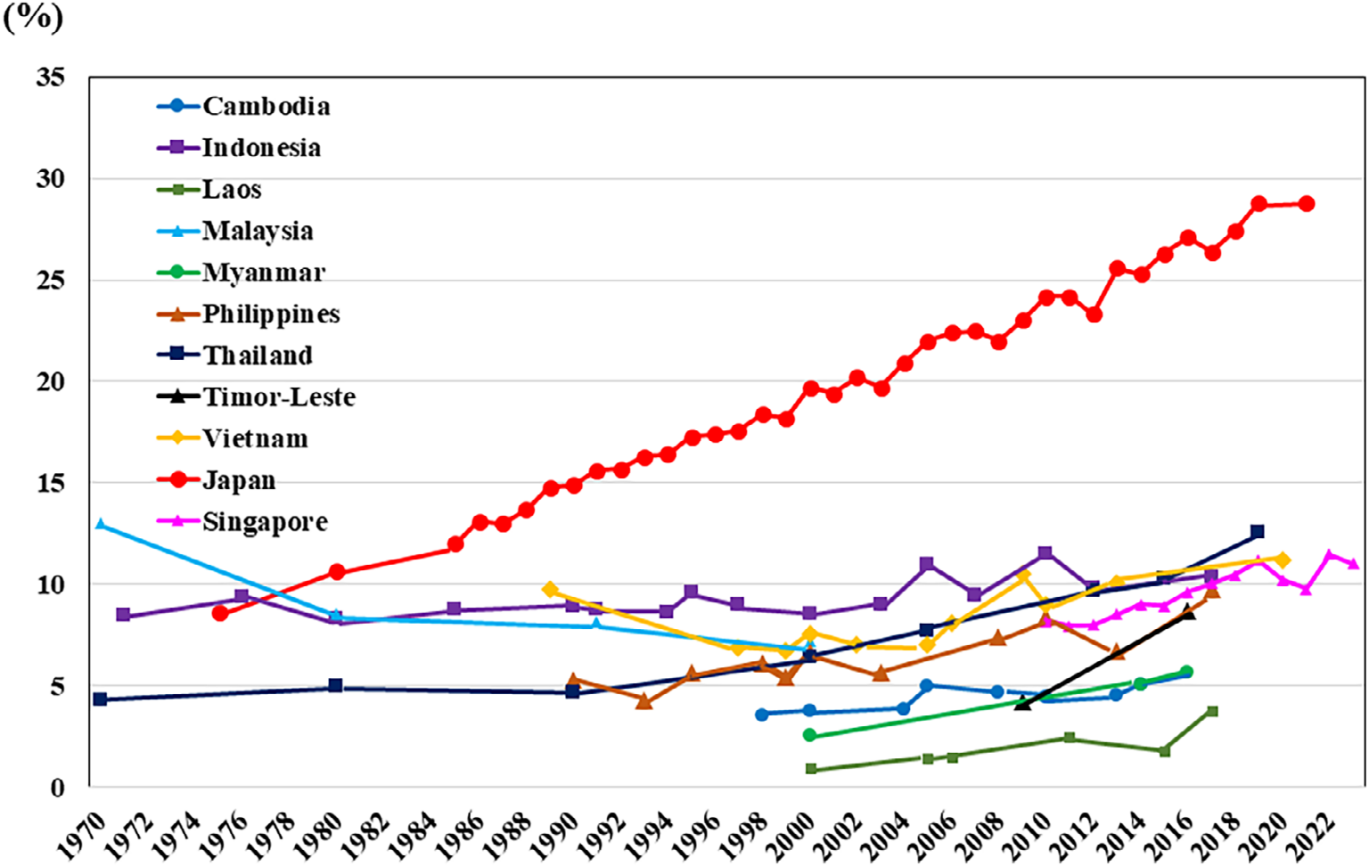
Trend in the percentage of people aged ≥65 years living alone. Data from United Nations, Department of Economic and Social Affairs, Population Division (2022). Database on the Households and Living Arrangements of Older Persons 2022 and Comprehensive Survey of Living Conditions 2023 by Ministry of Health, Labor and Welfare, the Japanese Government.

Importantly, the proportion of older adults living alone is steadily increasing in Japan (Fig. 19). On 28 August 2024, the Metropolitan Police Department reported that 28 330 individuals aged ≥65 years died alone at home nationwide in Japan during the 6‐month period from January to June 2024.^112^ It has been reported that living alone and reduced social activity are significant risk factors for loneliness among older adults.^113^, ^114^, ^115^ Conversely, social participation and being employed are considered protective factors against loneliness.^113^, ^115^, ^116^ Globally, Nordic countries reported an increasing retirement age and a high employment rate among older adults.^117^, ^118^ In 2020, Iceland, which has the highest employment rate for individuals aged 70–74 years among Nordic countries, recorded a rate of approximately 15%.^118^ Meanwhile, in 2020, Japan's employment rate for the same age group was 32.5%, more than twice that of Iceland, and has continued to rise.^119^

**Figure 20:**
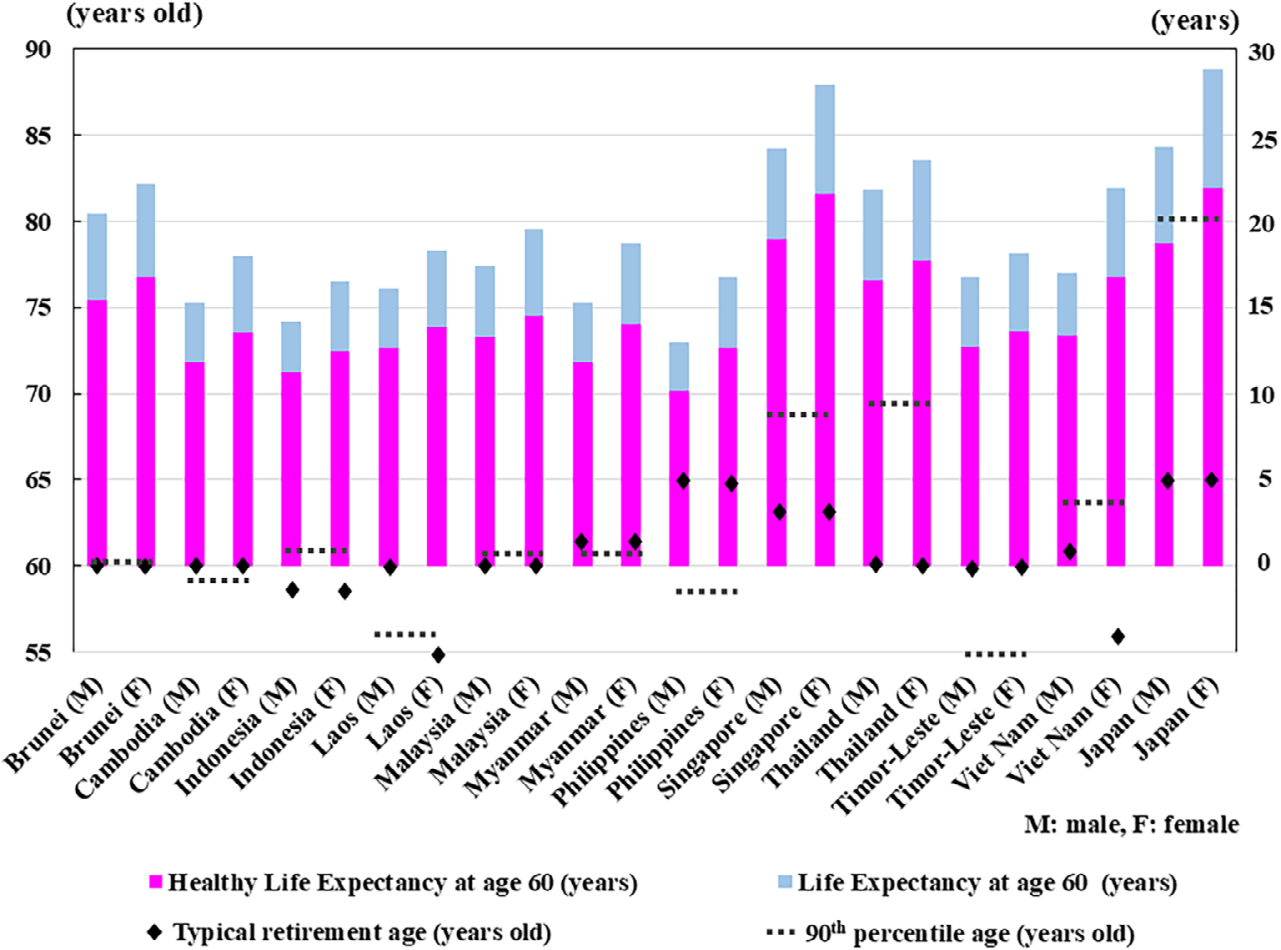
Healthy life expectancy at age 60 years, life expectancy at age 60 years, typical retirement age and 90th percentile age by country. Data from World Health Organization, The Global Health Observatory; United Nations, Department of Economic and Social Affairs, Population Division (2024). World Population Prospects 2024, Online Edition. The Labor Ministry of Thailand plans to raise the retirement age for both private and government sectors to 65 years.

There is still a large gap between the healthy life expectancy at age 60 years and retirement age in Southeast Asia and Japan (Fig. 20). This suggests that we humans might be stepping into uncharted territory. The health status of certain age groups in society varies from region to region and era to era, depending on the environment and culture. In fact, physical functions among older adults are improving in certain areas. As evidence, a longitudinal physical fitness study in Japan indicated that the walking function and flexibility of people aged 75–79 years exceeded that of people aged 65–69 years 25 years ago.^120^ The comprehensive geriatric assessments across countries support this assertion.^121^, ^122^, ^123^ Furthermore, the health status varies from person to person. It might be necessary to adopt measures tailored to the realities of each society in collaboration with the global community. In northeast Thailand, a program has been initiated to help older adults to farm sustainably on temple grounds, supported by young students.^124^ Although it is important to provide the appropriate care needed, it is also important not only to provide care, but also to respectfully encourage older people to take on the roles that are best for themselves and for society. Opportunities for interaction between young and older adults should be enhanced, allowing for the exchange of vitality and wisdom.

## Disclosure statement

The author declares no conflict of interest.

## Ethics statement

All procedures performed in this study were in accordance with the ethical standards of the institutional and/or national research committee, and with the 1964 Helsinki Declaration and its later amendments or comparable ethical standards.

## Data Availability

Data sharing is not applicable to this article as no new data were created or analyzed in this study.
